# Systems Biology of Immunomodulation for Post-Stroke Neuroplasticity: Multimodal Implications of Pharmacotherapy and Neurorehabilitation

**DOI:** 10.3389/fneur.2016.00094

**Published:** 2016-06-28

**Authors:** Mohammed Aftab Alam, V. P. Subramanyam Rallabandi, Prasun K. Roy

**Affiliations:** ^1^National Brain Research Centre, Gurgaon, India

**Keywords:** stroke, neuroprotection, rehabilitation, minocycline, direct current stimulation, transcranial magnetic stimulation

## Abstract

**Aims:**

Recent studies indicate that anti-inflammatory drugs, act as a double-edged sword, not only exacerbating secondary brain injury but also contributing to neurological recovery after stroke. Our aim is to explore whether there is a beneficial role for neuroprotection and functional recovery using anti-inflammatory drug along with neurorehabilitation therapy using transcranial direct current stimulation (tDCS) and repetitive transcranial magnetic stimulation (rTMS), so as to improve functional recovery after ischemic stroke.

**Methods:**

We develop a computational systems biology approach from preclinical data, using ordinary differential equations, to study the behavior of both phenotypes of microglia, such as M1 type (pro-inflammatory) vis-à-vis M2 type (anti-inflammatory) under anti-inflammatory drug action (minocycline). We explore whether pharmacological treatment along with cerebral stimulation using tDCS and rTMS is beneficial or not. We utilize the systems pathway analysis of minocycline in nuclear factor kappa beta (NF-κB) signaling and neurorehabilitation therapy using tDCS and rTMS that act through brain-derived neurotrophic factor (BDNF) and tropomyosin-related kinase B (TrkB) signaling pathways.

**Results:**

We demarcate the role of neuroinflammation and immunomodulation in post-stroke recovery, under minocycline activated-microglia and neuroprotection together with improved neurogenesis, synaptogenesis, and functional recovery under the action of rTMS or tDCS. We elucidate the feasibility of utilizing rTMS/tDCS to increase neuroprotection across the reperfusion stage during minocycline administration. We delineate that the signaling pathways of minocycline by modulation of inflammatory genes in NF-κB and proteins activated by tDCS and rTMS through BDNF, TrkB, and calmodulin kinase (CaMK) signaling. Utilizing systems biology approach, we show that the activation pathways for pharmacotherapy (minocycline) and neurorehabilitation (rTMS applied to ipsilesional cortex and tDCS) results into increased neuronal and synaptic activity that commonly occur through activation of *N*-methyl-d-aspartate receptors. We construe that considerable additive neuroprotection effect would be obtained and delayed reperfusion injury can be remedied, if one uses multimodal intervention of minocycline together with tDCS and rTMS.

**Conclusion:**

Additive beneficial effect is, thus, noticed for pharmacotherapy along with neurorehabilitation therapy, by maneuvering the dynamics of immunomodulation using anti-inflammatory drug and cerebral stimulation for augmenting the functional recovery after stroke, which may engender clinical applicability for enhancing plasticity, rehabilitation, and neurorestoration.

## Introduction

Recent investigations have reported that immune responses to inflammation are non-specific systemic infections associated with progression of neurodegenerative diseases via activation of macrophages (1). Minocycline is a tetracycline antibiotic having several properties, such as anti-inflammatory, anti-apoptosis, free radical scavenger, and protein misfolding (2). The therapeutic effects of minocycline in preclinical models of neurodegenerative diseases showed direct neuroprotection and reduction of microglial inflammatory responses (3). It has been reported in *in vivo* studies that minocycline blocks the adhesion of leukocytes to cerebrovascular endothelial cells induced by lipopolysaccharides, as well as tumor necrosis factor-α (TNF-α) production in the brain (4). *In vitro* studies have reported the anti-inflammatory effects of minocycline for neuroprotection (5) and in macrophages (6). Neuroprotective effects of minocycline include reduction of macrophage activation, prevention of the potentiation of ischemia-like injury to astrocytes and endothelial cells consolidating the brain tissue parenchyma (7). Although, the anti-inflammatory effects of minocycline are known to some extent, the direct effects of neuroprotection have not been well investigated in neurodegenerative diseases.

Several studies have shown that the physiological neuroprotection mechanisms that occur after stroke are targeted through various signaling pathways. Several studies suggest that the mechanisms associated with either reducing the size of infarct or enabling neurorestoration, involve the following entities: (i) anti-high mobility group box-1 activity (8); (ii) NF-κB (9); (iii) mammalian target of rapamycin (mTOR) inhibitor (10, 11); (iv) stimulation of toll-like receptors (TLR2 and TLR4) prior to brain ischemia (12, 13), (v) c-Jun N-terminal kinase (JNK) inhibitor (14); (vi) p38 mitogen-activated protein kinase (p38 MAPK) inhibitor (15); (vii) MEK1 pathway (16); (viii) MAPP/MEK/ERK inhibitor (17); and (ix) Minocycline-induced reduction of LPS-stimulated p38 MAPK activation, and stimulation of the phosphoinositide 3-kinase (PI3K)/Akt pathway (18).

Currently, little is known about endogenous counter regulatory immune mechanisms that can induce neurorestoration. The glycogen synthase kinase-3β (AKT/GSK-3β) pathway has been recognized as a protective pathway against cerebral ischemic injury. In cerebral ischemia models, it has been shown that remote limb conditioning does indeed activate and upregulate the pro-survival AKT pathway (19) and long-term protection against cerebral ischemia is afforded by limb post-conditioning that is associated with AKT, MAPK, phosphatidylinositol 3-kinase (PI3K), and protein kinase C (PKC) signaling pathways (20). NF-κB transcription factor family members, such as p50, p65/RelA in the hippocampus, are regulated by metabotropic glutamate receptor signaling and c-Rel transcription factor is responsible for the formation and maintenance of long-term memory (21). Minocycline directly inhibits matrix metalloproteinase (MMP)-9 activation through NF-κB pathway (22). *In silico* modeling of anti-inflammatory response has been reported for endotoxins (LPS) and corticosteroids by activating TLRs in NF-κB (23).

Taken together, the modulation of cell survival and death signaling by hypoxic/ischemic preconditioning appears to be capable of targeting multiple levels of signaling cascades. Several inhibitors targeted the point of convergence through distinct and interacting signaling pathways (crosstalk mechanism) for inflammation by activating macrophages that lead to neuroprotection. Also, cerebral stimulation-based transcranial magnetic stimulation and direct current stimulation enhances brain-derived neurotrophic factor (BDNF) and tropomyosin-related kinase B (TrkB) signaling (24, 25). In this study, we harness the convergent signaling pathways of pharmacotherapy (anti-inflammatory, immunomodulatory) and neurorehabilitation therapy (functional recovery) for efficient post-stroke neurorestoration by experimental and systems-level approach. We modeled using the systems biology approach of minocycline modulation of MMPs through NF-κB signaling pathway, a master regulator of inflammatory responses along with neurorehabilitation-based activation in BDNF and TrkB signaling.

## Materials and Methods

**Figure 1:**
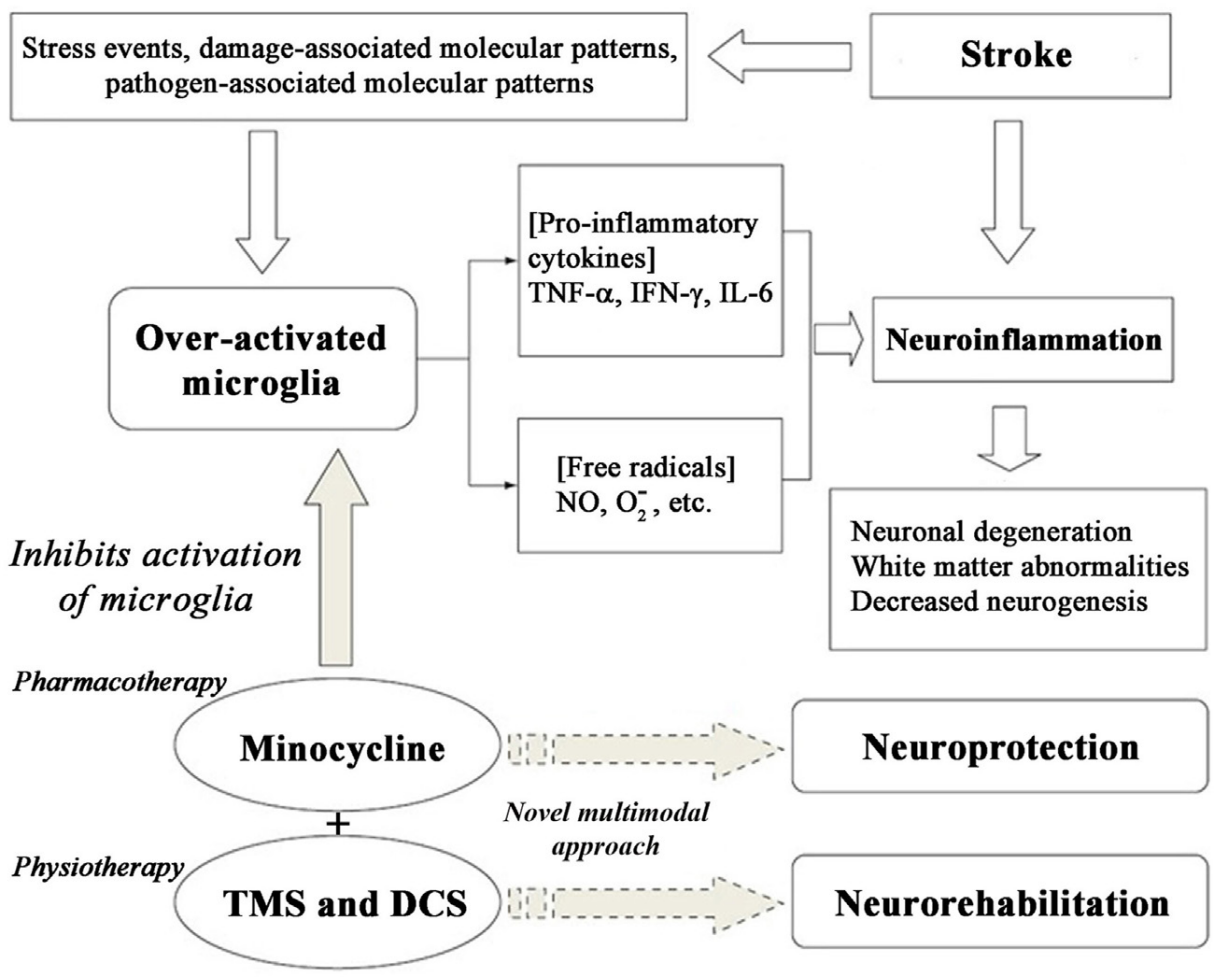
**Cascade of cell signaling events post-stroke and enabling neuroprotection using minocycline**.

There are numerous cellular responses, stress events, neuronal death, and inflammatory mechanisms, including over-activation of microglia as shown in Figure 1. We utilized and targeted anti-inflammatory drug in our approach. We formulated various cell kinetics interactions utilizing the systems biology platform for pharmacotherapy (Module 1) and neurorehabilitation (Module 2)-based activation through different signaling pathways, such as NF-κB, BDNF, and TrkB. To formulate and model the effect of therapeutic interventions, we activated the corresponding signal transduction factor accordingly. For minocycline, activation input was in the form of a constant single impulse or step function, while for the transcranial direct current stimulation (tDCS) and repetitive transcranial magnetic stimulation (rTMS) inputs, we used repeated series of electrical or magnetic pulses involved in cerebral stimulation. The chemical reactions of the modules were constructed using the framework of ordinary differential equations (ODEs), which were then solved by numerical computation using the Runge–Kutta method.

### Pharmacotherapy (Minocycline) through NF-κB Signaling Pathway

**Figure 2:**
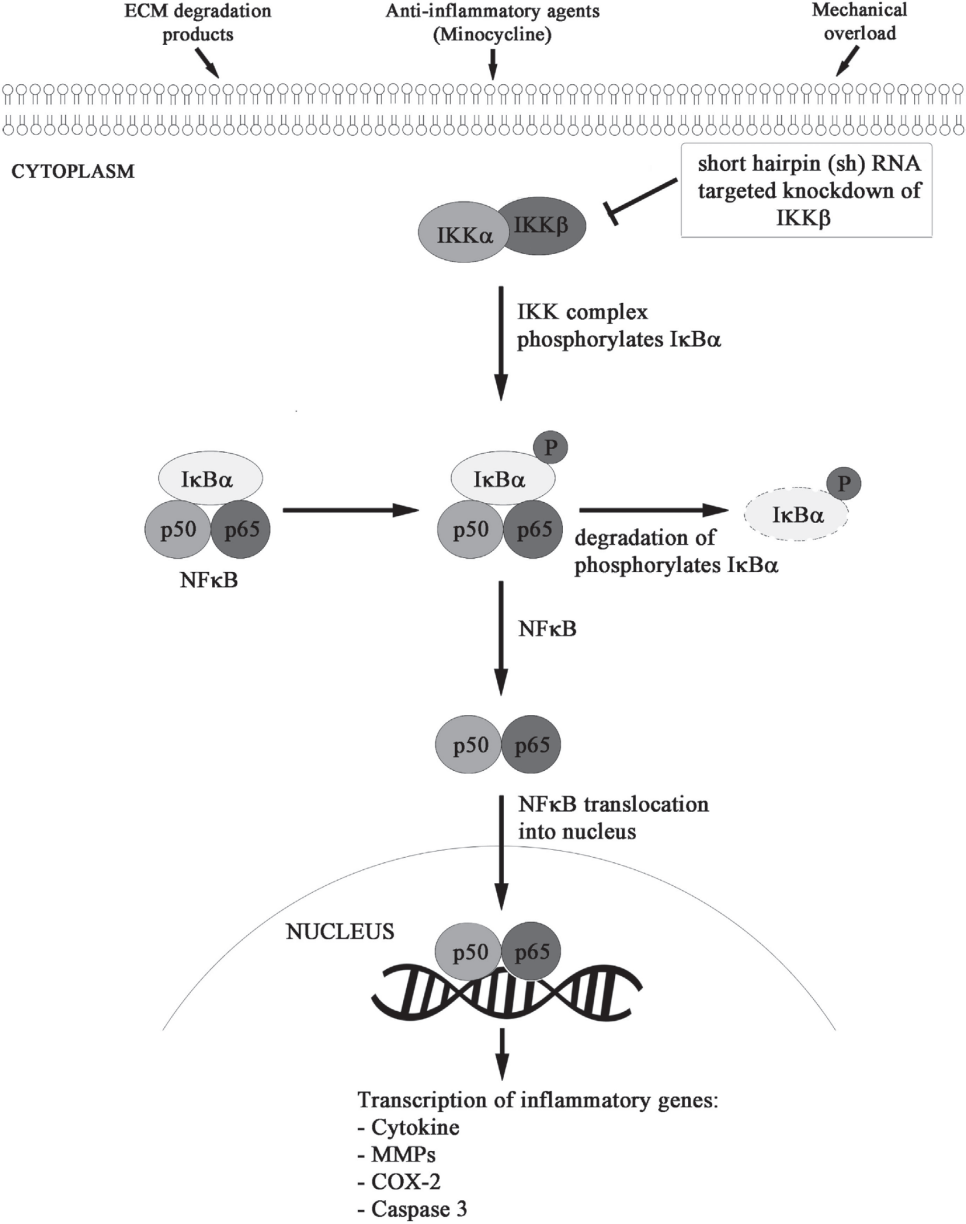
**Minocycline-activated anti-inflammatory response through NF-κB signaling pathway**.

We formulated and elucidated the cascade of cell signaling events using ODEs that were solved using ODE23 solver in MatlabR2013a. We modeled the inflammatory mechanism of minocycline through regulation of genes and proteins that significantly corresponded to NF-κB transcriptome as shown in Figure 2. Studies have suggested that minocycline-induced suppression of NF-κB activity is mediated by the inhibition of M1 microglia and activation of M2 microglia. All the sequential chemical processes are explained in Module 1. The *in vitro* and *in vivo* studies have shown that both the acute and chronic doses of minocycline lead to suppression of p65 phosphorylation and nuclear translocation accompanied by downregulation of NF-κB activity and endogenous MMP9 protein levels (22).

Using the dynamics of known NF-κB stages (26, 27), the action of minocycline via the signaling pathway was represented as shown in Module 1. The sequence depicts a chronological order of the formation of different biochemical species (mentioned on the right side after % symbol) with their corresponding reaction kinetic equation (on the left side).

The non-zero initial concentration of the chemical species and the association and dissociation rate constants are taken from previous experiments (23, 26) given in APPENDIX (Table A1). The ODE equations shown in Module 1 were solved using ODE23 solver in MatlabR2013a.

### Neurorehabilitation Therapy through BDNF and TrkB Signaling Pathway

To elucidate the process of neurorehabilitation therapy using tDCS and rTMS via BDNF and TrkB pathway. We constructed the upstream and downstream dynamics of the pathway using currently available data (28). The corresponding chemical reactions are formulated as ODE and shown in Module 2.

#### Cerebral Stimulation (rTMS and tDCS) through BDNF and TrKB

**Figure 3:**
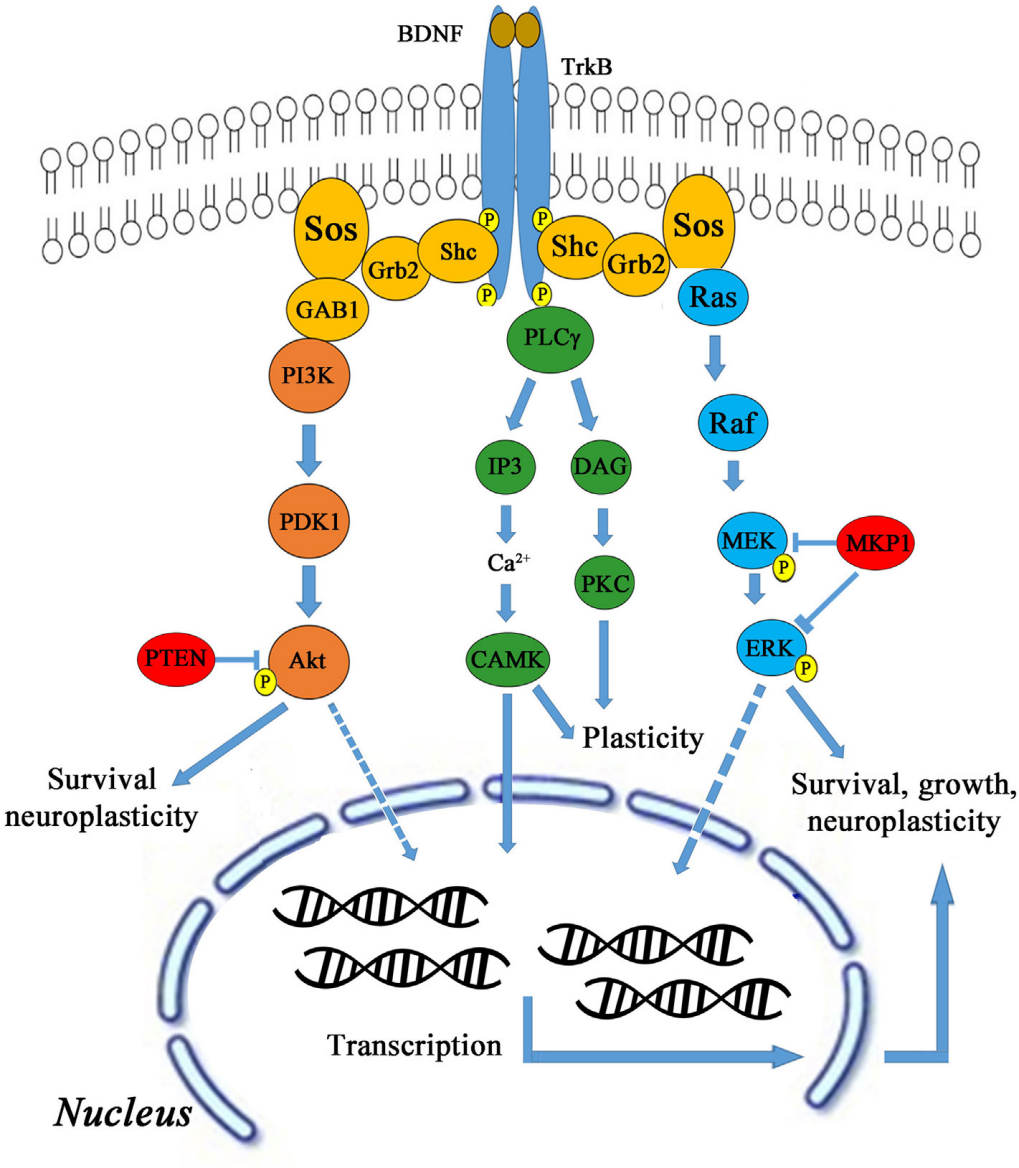
**BDNF and TrkB signaling pathway**.

Brain-derived neurotrophic factor activates TrkB through several downstream signaling pathways, such as AKT, CaMK, Ras/Raf/MEK/ERK leading to cell survival, growth, and neuroplasticity as shown in Figure 3. BDNF activates TrkB stimulation via phosphatidylinositol-4,5-bisphosphate 3-kinase (PI3K) and also activates proteins like Shc, Grb-2, and Gab-1. The PI3K is also activated by binding to Ras homolog enriched by brain glutamine triphosphate (Ras-GTP). TMS was delivered to male Sprague Dawley rats using 1600 stimuli at 5 Hz in four blocks of each 400 stimuli in 2.5 min with 1 min inter-block interval (24). Anodal tDCS was applied with current density of 0.04 mA/cm^2^, total charge of 0.048 C/cm^2^ for 20 min (25). Activated PI3K phosphorylates PtdIns[3,4]P2 (PIP2) and PtdIns[3,4,5] P3 (PIP3), and then PIP3 is dephosphorylated by phosphotase and tensin homolog (PTEN). PIP3 activates AKT (also known as protein kinase B) leading to the formation of Rheb-GTP, a regulator of rapamycin (TOR) (29, 30). AKT needs phosphorylation twice to become active. PIP3 binds to AKT and recruits it in the membrane and does the same for Pyruvate dehydrogenase lipoamide kinase isozyme 1 (PDK1).

In the membrane, PDK1–PIP3 complex phosphorylates AKT on Thr308 and further phosphorylates on Ser-473 by PIP3-PDK2. Both these phosphorylates are reversed by protein phosphatase 2A (PP2A). The doubly phosphorylated AKT becomes active and phosphorylates tuberous sclerosis 1,2 (TSC1–TSC2), which in turn regulates Rheb-GTP levels. The unphosphorylated complex has GTPase-activating protein (GAP)-type properties toward Rheb, while TSC1–TSC2 hydrolyzes Rheb-GTP to Rheb-GDP (31). The active AKT inactivates TSC1–TSC2 and only Rheb-GTP remains. Therefore, the net effect is, PIP3 activates AKT leading to activation of downstream target TOR. Furthermore, Rheb-GTP activates TOR and the converged input from MAPK leads to S6K activation, forming active 40S for translation (32, 33). The Rheb-GTP binds to the TOR_complex to stimulate kinase activity through phosphorylation in each kinase, viz. MAPK, TOR_complex, and PDK1. Next, dephosphorylation occurs at all the sites by PP2A. The double- and triple-phosphorylated S6K becomes active and phosphorylates S6, which is a subunit of 40S ribosomal protein.

Another possible signaling mechanism is phospholipase C gamma (PLC-γ) that in turn cleaves PIP2, diacylglycerol (DAG), and inositol trisphosphate (IP3). DAG remains bound to membrane, whereas IP3 is released as a soluble structure into the cytosol. Then IP3 diffuses through cytosol and binds to IP3 receptors, particularly calcium channels in a smooth endoplasmic reticulum, and then activates Ca^2+^ in calmodulin kinase (CaMK) activity. Correspondingly, DAG activates PKC for plasticity (34). CaMKIII is activated by Ca^2+^ and inactivated by S6K. CaMKIII inhibits eukaryotic elongation factor 2 (eEF2), the net effect of Ca^2+^ on elongation factor is inhibitory and of S6K is excitatory. eEF2 is the major substrate for CaMKIII. CaMKIII catalyzes eEF2 phosphorylation at thr-56 and thr-58, strictly in the presence of Ca^2+^ and CaM (35). CaMKIII phosphorylates eEF2 to render it inactive. Dephosphorylation of eEF2 by PP2A restores its activity.

The S6K phosphorylates CaMKIII at Ser-366 and decreases its activity and thereby increases the level of eEF2 (36). Calmodulin dependence of CaMKIII activity was measured in terms of eEF2 phosphorylation (37). Next, the active S6K is added to CaMKIII and time course of CaMKIII is measured. Then, active eEF2-thr-36 is measured with respect to time. The dose–response for 40S complex is measured for different 40S concentrations (38), the dephosphorylation reaction and the formation of phosphorylated eEF2 (28). The 40S and eEF2 bind to form the translation complex, leading to protein synthesis. MAPK is downstream of PI3K as well as CaM-Ca4. The MAPK activity is closely related to synaptic activity leading to Ca^2+^ influx. The alternate mechanism is through downstream signaling of Ras/Raf/MEK/ERK pathway for cell survival, growth, and neuroplasticity.

The non-zero initial concentration of chemical species is given in APPENDIX (Table A2). All these parameters are taken from the experimental studies related to memory, learning, and synaptic plasticity through BDNF and TrkB signaling. The implications of tDCS and rTMS in the present study are to enhance the post-stroke motor-learning along with functional recovery in ischemic stroke patients. The association and dissociation rate constants are taken from experimental studies as given in APPENDIX (Table A3). The aforesaid equations are given in Module 2, which were solved by ODE23 solver in MatlabR2013a.

## Results

We simulated the biochemical processes of both Modules 1 and 2. Our observations and inferences are explained in details below.

### Pharmacotherapy (Minocycline)

**Figure 4:**
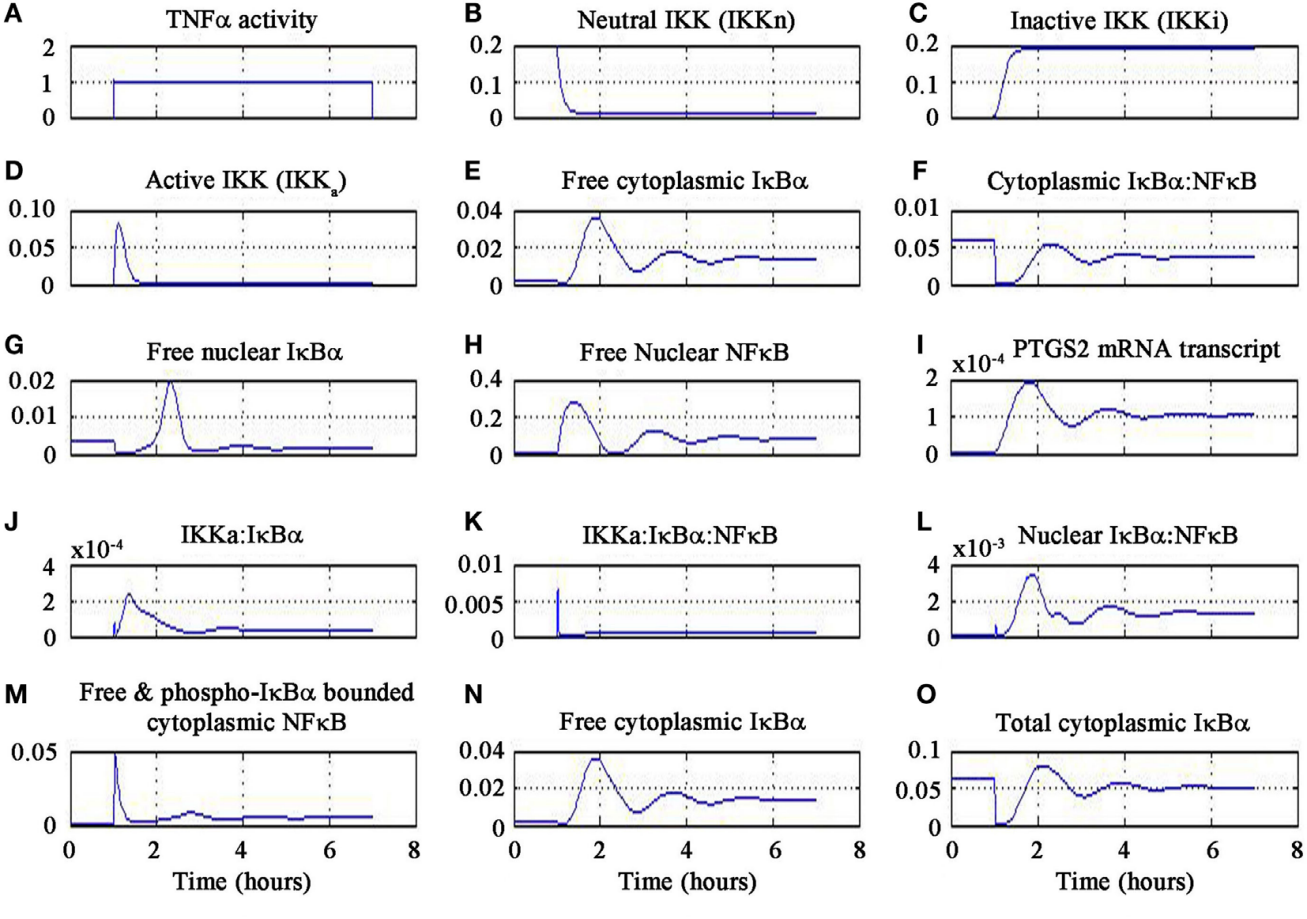
**Minocycline-induced TNF-α activity in NF-κB pathway**.

The results of the signaling activation by the pharmacological agent minocycline are shown in Figure 4. A sub-optimal dose of 4.5 mg kg^−1^ was chosen from a dose escalation study of minocycline in stroke patients (81). Our findings delineate the inflammatory mechanism of minocycline through regulation of genes and proteins that correspond to the NF-κB transcriptome. Our simulation has shown the molecular mechanism of minocycline that could be attributed to modulation of NF-κB signaling. Minocycline suppressed NF-κB activation in neurons and glial cells and was correlated with attenuation of IκBα kinase (IKK) activation, IκBα phosphorylation and degradation, and p65 phosphorylation and nuclear translocation. The inhibition of IKK was found to be associated with suppression of activated-microglia (neurotoxic M1 microglia) and correspondingly enhanced the restorative microglia (neuroprotective M2 microglia). Furthermore, the simulation demonstrated that minocycline upregulated TNF-α expression. Enforced TNF-α expression induced NF-κB activity and minocycline rescued through inhibition of iNOS and NO production in cells. Our simulated results are consistent with the experimental findings that suppressed NF-κB activation and abrogated the inhibitory effect of minocycline on the transcription factor, TNF-α. These results suggest that minocycline led to suppression of p65 phosphorylation and nuclear translocation accompanied by downregulation of NF-κB activity and endogenous MMP9 protein levels and its target, PTGS2 gene.

In the simulation, a minocycline dose of 4.5 mg kg^−1^ induced TNF-α activity for 6 h as shown in Figure 4A. The neutral IKK activity gradually reduced from 60 to 90 min and then asymptotically saturated downwards to baseline as shown in Figure 4B. Similarly, the inactive form of IKK increased from 60 to 90 min and then saturated to baseline as shown in Figure 4C. The active IKK expression increased from 60 to 90 min, peaked at 90 min and then fell gradually till 120 min and saturated downwards to basal level as shown in Figure 4D. Free cytoplasmic IκBα increased from 60 to 90 min, peaked at 120 min and then gradually fell till 180 min and oscillated as shown in Figure 4E. The cytoplasmic IκBα|NF-κB activity initially fell till 60 min, then peaked at 120 min, fell down till 180 min, and then showed oscillatory behavior as shown in Figure 4F. The free nuclear IκBα increased from 60 to 140 min, fell down till 180 min and finally damped as in Figure 4G. The free nuclear NF-κB activity increased from 60 to 90 min, fell down from 90 to 120 min, and then showed oscillatory behavior as in Figure 4H.

The PTGS2 gene transcription increased from 60 to 120 min, fell down till 180 min, and then oscillated as shown in Figure 4I. The expression of IKKa|IκBα complex increased from 60 to 90 min, gradually fell till 180 min, and then oscillated as in Figure 4J. The expression of IKKa|IκBα|NF-κB complex peaked at 60 min and then fell afterwards as shown in Figure 4K. The nuclear IκBα|NF-κB expression increased from 60 to 120 min and then gradually fell till 180 min and oscillated as in Figure 4L. Free and phosphorylated IκBα bounded to cytoplasmic NF-κB peaked at 60 min, fell gradually till 90 min and damped as in Figure 4M. Free cytoplasmic IκBα expression increased from 60 to 120 min then gradually fell till 180 min, and finally oscillated as in Figure 4N. The total cytoplasmic IκBα activity initially fell down till 60 min and then onward gradually increased till 120 min, and finally showed oscillatory behavior as in Figure 4O. It is important to note that the temporal profile for the chemical species will not change for different concentrations of minocycline.

### Neurorehabilitation Therapy Using tDCS and rTMS

**Figure 5:**
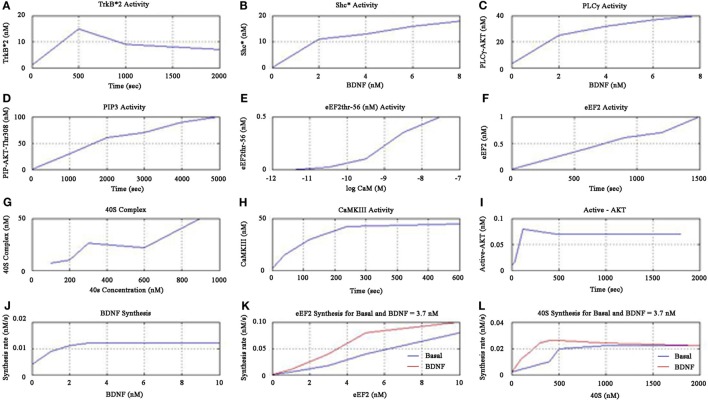
**Cerebral stimulation induced BDNF and TrkB signaling**.

The simulations showed increased activity of TrkB*2 from 0 to 500 s, and then fell till 1000 s, and then asymptotically saturated downwards to a definitive level as shown in Figure 5A. The Shc* activity increased linearly when plotted against BDNF till the BDNF concentration reached 2 nM as shown in Figure 5B. The PLC-γ activity increased linearly when plotted against BDNF till the concentration of 3.7 nM as in Figure 5C. Furthermore, the PIP3 activity linearly increased as shown in Figure 5D, while the cell elongation factor (eEF2) eEF2thr-56 showed sigmoid relationship when plotted against low concentrations of the calcium-binding protein Calmodulin, CaM (measured in terms of negative log) as in Figure 5E. eEF2 activity was observed to linearly increase with time as in Figure 5F. We observed that 40S complex activity displayed a declining sigmoidal function with an increased activity till 300 nM when plotted against 40S concentration, followed by constant activity (very slowly declining) from 300 to 600 nM, and thereafter finally the activity rises beyond 600 nM as in Figure 5G.

Continuing the formulation further, we note that the kinase CaMKIII activity increased till 240 s and then saturated upwards as shown in Figure 5H. We found active AKT increase till 100 s, decreased after 480 s and then downward saturation to baseline as in Figure 5I. We inferred that the BDNF synthesis rate was maximum for the concentration of 3.7 nM as shown in Figure 5J. Moreover, we noticed eEF2 synthesis rate linearly increased when plotted against eEF2 concentration, for both basal kinase activity and for BDNF concentration of 3.7 nM (Figure 5K). We found that the synthesis rate of eEF2 for BDNF concentration of 3.7 nM was double the basal kinase level, when the eEF2 concentration is 5 nM as shown in Figure 5K. We also showed that the 40S synthesis rate peaked at 500 nM of 40S concentration for both basal kinase level and for BDNF level of 3.7 nM, and 40S synthesis rate was higher for BDNF level of 3.7 nM than that for basal kinase level, as shown in Figure 5L.

**Figure 6:**
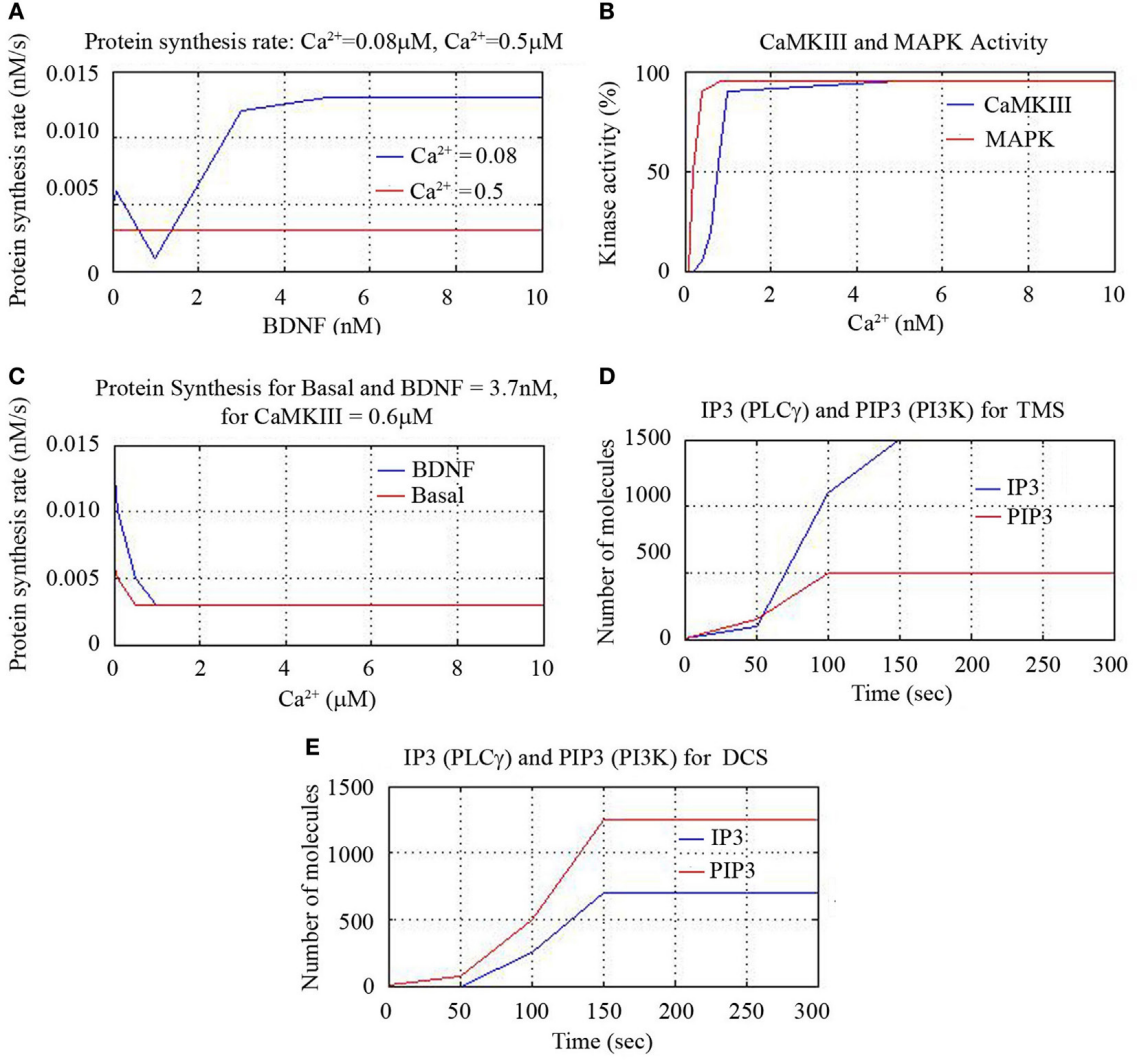
**Cerebral stimulation induced IP3, PIP3, and CaMKIII activity**.

We also found that protein synthesis rate is constant for all BDNF concentrations for Ca^2+^ = 0.5 nM and notice that increase of synthesis rate occurs beyond 0.1 nM concentration for Ca^2+^ = 0.08 nM. This suggests that BDNF = 3.7 nM and lower Ca^2+^ concentration leads to higher protein synthesis rate as shown in Figure 6A. We plotted the percentage of kinase enzymes CaMKIII and MAPK activity against Ca^2+^ concentration and found MAPK activity peaked for Ca^2+^ = 0.5 nM, while CaMK activity peaked for Ca^2+^ = 1.0 nM as shown in Figure 6B. The protein synthesis rate for constant CamKIII = 0.6 μM gradually decreased for both basal kinase activity and for BDNF level of 3.7 nM, till Ca^2+^ = 0.1 μM. Thereafter, the protein synthesis activity was constant for higher Ca^2+^ concentrations. However, the synthesis rate was initially higher for BDNF level of 3.7 nM than for basal kinase activity, for lower values of Ca^2+^ concentration (<0.1 μM), as in Figure 6C.

As mentioned earlier, we activated the corresponding signal transduction factor accordingly (inositol triphosphate IP3, and the related compound, phosphatidyl-IP3, viz. PIP3). First, the initial activation of IP3 through phospholipase C (PLC-γ) and the activation of PIP3 through PI3K were plotted for TMS and DCS. TMS showed higher level of IP3 than PIP3 with higher number of reactant molecules generated till 150 s, and then the number of molecules saturated to a definitive level as in Figure 6D. DCS showed higher PIP3 than IP3 with an increased number of molecules till 150 s as in Figure 6E.

Thus, we have shown that both TMS and DCS activated CaMKIII that shows the cell survival and synaptic plasticity. To draw a perspective, we elucidated that all the modalities (minocycline, tDCS, rTMS) have different pathways with common activation of *N*-methyl-d-aspartate (NMDA) receptors. Therefore, pharmacotherapy and neurorehabilitation can be incisively combined for additive beneficial effect.

## Discussion

Oxidative stress and inflammation are the two major pathophysiological mechanisms involved during early and late phases of ischemic stroke. Brain tissue is not armed with antioxidant defenses, so reactive oxygen species and other free radicals/oxidants released by inflammatory cells, threaten tissue viability in the core ischemic region. This study targets the molecular aspects of inflammation in ischemic stroke and offers potential therapeutic strategies that target neuroinflammation and modulates the innate immune system. In addition to antibiotic activity, minocycline exhibits anti-inflammatory responses in both monocytes and macrophages. Earlier studies have reported that minocycline causes significant reduction in the inflammatory response of LPS-challenged monocytes, reducing LPS-induced transcription of pro-inflammatory TNF-α, interleukin-1 beta (IL-1β) (82), interleukin-6 (IL-6), and cyclooxygenase-2 (COX-2) (83), the LPS stimulated TNF-α, IL-6, and PGE2 release. Minocycline inhibited LPS-induced activation of the lectin-like oxidized low-density lipoprotein receptor-1 (LOX-1), NF-κB, LPS-induced TNF-α factor (LITAF) and the Nur77 nuclear receptor (20). It has been reported that the neuroprotective effects of minocycline are associated with inhibition of iNOS induction and NO production in glial cells, which are mediated by the LPS-induced production of TNF-α (84).

### Pharmacotherapy: Minocycline

The regulation of inflammatory genes and proteins in NF-κB signaling pathway are MMPs and Cox-2. MMPs are a family of zinc-dependent proteases responsible for the degradation of extracellular matrix proteins and capable of causing bioactive molecules. Cox-2 inhibitors are non-steroidal anti-inflammatory drugs (NSAIDs). The *in vitro* models showed direct neuroprotection by minocycline as well as inhibition of other inflammatory mediators, such as iNOS, MMPs, TNF-α, and tissue plasminogen activator (tPA). However, some earlier studies have reported expression of inflammatory cytokines, such as TNF-α in ischemic neurons (85) and tPA (86), and generation of neuronal tPA has been thought to mediate microglial activation. Thus, these studies suggested that neurons may be capable of secreting inflammatory mediators themselves from classical immune cells, including microglia and other glial cells, such as astrocytes. Also the neuronal survival depends on the endogenous or exogenous tPA, which can be either neuroprotective or neurotoxic. Similar conclusions could be made of neuronal generation of iNOS and MMPs. MMPs 2 and 9 levels are elevated a few hours after ischemia (87) and maintain increased activity for days after the onset of stroke (88). The specific class of agents inhibits these enzymes, reverts the breakdown of laminin (89), and prevents increased barrier permeability, edema, and hemorrhage after ischemic stroke (90).

Ischemic stroke activates a complex cascade of events and tetracycline antibiotics may exert anti-inflammatory effects by multiple mechanisms. The models of brain injury, which involve matrix degradation and vascular instability, targets inhibition of proteolytic cascade. However, there are limitations for the usage of MMP inhibitors as therapeutic agents because of their poor solubility. Minocycline has been shown to be a neuroprotective agent by inhibiting MMP2 and MMP9 activation by ischemia (91). The dysregulation of the proteolytic cascade at the level of the endothelial and microglial cells is one of the common pathological mechanisms of ischemic brain damage. Minocycline targets through the interference of this cascade and might be the key pathway for neuroprotection and salvage of injured tissue. Animal models of permanent cerebral ischemia showed that minocycline treatment was protective and beneficial in cerebral ischemia by mechanisms that are not linked to the production of free radicals during reperfusion. Understanding inflammatory interactions of all brain cell types is needed to identify how they may be manipulated to provide neuroprotection. Studies of minocycline effects on brain cells in addition to microglia are timely, as minocycline has shown efficacy in clinical studies for various neurodegenerative disorders (92, 93) and ischemic stroke (81, 94, 95). Thus, further investigations are needed for a better understanding of the complexities of immune responses of various types of cell bodies following brain injury.

### Direct Current Stimulation

The primary mechanism of tDCS for inducing cortical excitability shifts is a sub-threshold modulation of the neuronal resting membrane potential. The anodal stimulation outcome is a sub-threshold depolarization, while cathodal stimulation hyperpolarizes neuronal membranes. During a short tDCS, which elicits no after-effects (stimulation duration of about 4 s), synaptic mechanisms are not involved, as shown by the absence of effect of NMDA receptor antagonists, the GABA agonist lorazepam and the monoamine reuptake blocker amphetamine on the tDCS-induced excitability changes under these conditions. Furthermore, the after-effects are not due to reverberating electrical circuits or other purely electrical phenomena, as shown in early animal experiments. They depend on modifications of NMDA receptor efficacy, since these are blocked by the NMDA receptor antagonist dextromethorphan, but prolonged by the partial NMDA receptor agonist d-cycloserine (96). The tDCS polarity-dependent shift of NMDA receptor function seems to be initiated by the respective membrane potential shift and probably by the accompanying cortical activity modification, because it is prevented by the sodium channel blocker (carbamazepine). Likewise, tDCS requires the neurophysiological experimental studies for the selection of electrode montage in stroke (97). Furthermore, *in silico* modeling studies are required to understand the neuromodulatory effects of tDCS and helps in determining the optimal levels of anodal and cathodal stimulation that is beneficial for stroke patients.

Earlier studies also reported a decrease in IP 3 receptor mRNA level in the cortex at 4 h after a 45 min ischemic insult; however, lesser change was noticed for IP3 3-kinase mRNA levels during the initial 8 h after reperfusion (98). Also, IP3 receptor mRNA level in the peri-infarct areas was also reduced, specifically at a delayed time of reperfusion. The staining of cortical neurons in the infarct area showed morphological changes between 4 and 8 h after reperfusion (99). Thus, the decrease in IP3 receptor mRNA level probably occurred at a time prior to alteration of neuron morphology. A recent study showed that DCS stimulation-induced vasodilation occurs without IP3 receptor activation (100).

Brain-derived neurotrophic factor induces the transformation of early- to late-phase long-term potentiation (LTP) in the presence of protein synthesis inhibitors and BDNF–TrkB signaling is involved in synaptic tagging (101). BDNF activates the RAS/RAF signaling pathway and produces ERK, promoting gene transcription through cAMP-response element binding protein (CREB) in the cell nucleus. NMDA activation on the cell membrane produces a calcium influx that activates calcium CaMK IV, phosphorylating glycogen synthase kinase 3B (GSK3B) and allowing B-catenin to activate CREB. Recent studies have provided different mechanistic explanation for the early and late phases of LTP. The mechanism for later phases of LTP required for memory storage is the complex of CaMK signaling with the NMDA receptor (102). Furthermore, neurophysiological and *in silico* modeling studies are required to understand the neuromodulating effects of tDCS, and help in determining the optimal levels of anodal and cathodal stimulation that is beneficial for stroke patients.

### Transcranial Magnetic Stimulation

Earlier study have reported the effects of rTMS and illustrated the mechanisms of rTMS in regulating cognitive capacity. rTMS enhances spatial memory behavior, neuron and synapse morphology in the hippocampus, and synaptic protein markers and BDNF/TrkB in normal aging mice (103). Aging mice inhibited the activation of BDNF–TrkB signaling pathway and showed hippocampal-dependent cognitive impairment in relative to adult animals, with the reduced transcription and expression of synaptic protein markers, such as growth-associated protein 43 (GAP43), synaptophysin (SYN), post-synaptic density protein 95 (PSD95), including decreased synapse density, and PSD thickness. Surprisingly, low-intensity rTMS (110% average resting motor threshold intensity, 1 Hz) triggered the activation of BDNF and TrkB, upregulated the level of synaptic protein markers, increased synapse density and PSD thickness, and reversed the spatial cognition dysfunction in aging mice. On the other hand, high-intensity rTMS (150% average resting motor threshold intensity, 1 Hz) was harmful, inducing reduction of PSDs thickness, disordered synaptic structure, as well as reduction in the number of synapses, and downregulation of BDNF–TrkB and synaptic proteins. The aging-induced cognitive deficits depends on the intensity level of TMS, which are closely associated with hippocampal structural synaptic plasticity that plays an important role in regulating cognitive behavior via changing structural synaptic plasticity through BDNF signaling. The effect of rTMS on functional recovery and its underlying molecular mechanism has been studied by assessing proteins associated with neural plasticity in a sub-acute ischemic rat model (104). A total of 3,500 impulses with 10 Hz frequency were applied to the ipsilesional cortex from post-operative day 4 over a 2-week period. The study reported that rTMS group showed more functional improvement on the beam balance test and the immunohistochemistry analysis showed stronger Bcl-2 and weaker Bax expression when compared with the sham group and noticed no significant difference in the expressions of NMDA and MAP-2 (104). However, rTMS may increase or decrease motor cortical excitability mostly depending on the characteristics of the stimulation protocol (105). Non-invasive cortical stimulation upregulates excitability in M1 lesioned hemisphere and downregulates in M1 intact hemisphere that could contribute to correcting abnormalities in inter-hemispheric inhibition identified after stroke (106). Thus, both the pharmacotherapy and neurorehabilitation therapy converge through the NMDA receptor activation.

One of the challenges for antibiotic therapy is the need to identify the optimum dose level. Recently, a reliable and feasible approach of dose estimation using chemical binding kinetics was proposed by Abel et al. (107). Another challenge is determining the impact of combinational multiple drug therapies on several proteins. A recent study suggested an approach using linear superposition of their responses to individual therapy (108). Utilizing these approaches, we can develop *in silico* models that could provide insight into the design of clinical trials of immunomodulatory therapies, ranging from optimal patient-specific dose selection, and duration of proposed therapeutic interventions (109).

## Conclusion

In this paper, we utilize a systems biology approach to maneuver the dynamics of immunomodulation using linear combination of doses of pharmacotherapy (minocycline) in NF-κB signaling pathway along with adjuvant neurorehabilitation therapy (rTMS or tDCS) through BDNF and TrkB signaling pathway stimulation for enhancing synaptic plasticity, rehabilitation, and neurorestoration. Thus, a thorough understanding and modeling of pathways, along with the optimal therapeutic doses could lead us to prevent cell death at an earlier stage and with a higher chance of maintaining the long-term viability of the cell protection after stroke. To conclude, further studies are required to dissect, report, and analyze these pathways toward the goal of improving neurorestoration by multimodal therapy for stroke and related neurovascular disorders.

## Author Contributions

The research was conceived and planned by PR, jointly with inputs from MA and VR. The numerical experimentation was performed by VR and the biological studies investigated by MA. All authors examined and evaluated the data. VR, MA, and PR wrote the manuscript. All authors read and approved the final manuscript and have no conflict of interests.

## Conflict of Interest Statement

The authors declare that the research was conducted in the absence of any commercial or financial relationships that could be construed as a potential conflict of interest.

## Appendix

**Table A1:** **Chemical species for Module 1**.

**x(1)**	IKKn neutral
**x(2)**	IKKa active
**x(3)**	IKKi inactive
**x(4)**	(IKKa|IkBa)
**x(5)**	(IKKa|IkBa|NFkB)
**x(6)**	NFkB
**x(7)**	NFkBn
**x(8)**	PTGS2
**x(9)**	PTGS2 transcription
**x(10)**	IkBa
**x(11)**	IkBan
**x(12)**	IkBat
**x(13)**	(IkBa|NFkB) cytoplasmic
**x(14)**	(IkBan|NFkBn) nuclear
**x(15)**	Control early gene

**Table A2:** **The following association and dissociation rate constants for module I are taken from Ref. (23, 26)**.

Constant	Description
TN = 0/1 (OFF/ON state)	TNF-alpha inactive/active
c1c = 0.00005/100	Inducible transcription (control gene)
c2c = 0	Constitutive transcription (control gene)
c3c = 0.0004	mRNa degradation (control gene)
e1a = 0.0005	IkBa nuclear export Hoff, Fitted
i1a = 0.001	IkBa nuclear import Hoff, Fitted
e2a = 0.01	(IkBa|NFkB) nuclear export, Hoff blue (any short)
i1 = 0.0025	NFkB nuclear import, Hoff blue (short correspond to a1)
c1a = AA*0.00005/100	Inducible (linear) IkBa mRNA synthesis, Fitted
c2a = AA*0.000000	Constitutive mRNA IkBasynthesis Fitted
c3a = 0.0004	mRNA IkBa degradation, Fitted
c4a = 0.005*100	IkBa translation rate, Fitted
c5a = 0.0001	IkBa degradation rate, Pando
c6a = 0.00002	(IkBa|NFkB) degradation, Hoff
AA = 1	AA = 1 wild-type cell, AA = 0 IkBa-deficient cell
t1 = 0.1	Degradation of (IKK|IkBa) (any short)
t2 = 0.1	Degradation of (IKK|IkBa|NFkB) (any short)
a1 = 0.5	IkBA*NFkB association Hoff (short, correspond to i1)
a2 = 0.2	IKK*IkBa association, Fitted
a3 = 1	IKK*(IkBa|NFkb) association, Fitted
kdeg = 0.000125	Degradation of IKKa, IKKn, and IKKi
kprod = 0.000025	IKKn production rate
r3 = 0.0015	Spontaneous inactivation, Fitted
r2 = 0.1	Inactivation caused by PTGS2, Fitted
r1 = 0.0025	Activation caused by drug, Fitted
c5 = 0.0003	PTGS2 degradation rate, IkBa*5
c4 = 0.005*100	PTGS2 translation rate (Assumed)
c3 = 0.0004	PTGS2 mRNA degradation rate (Assumed)
c2 = AB*0.00000	constitutive PTGS2 mRNA synthesis (Assumed)
c1 = AB*0.00005/100	inducible PTGS2mRNA synthesis (Assumed)
AB = 1	AB = 1 wild-type cell, AB = 0 PTGS2-deficient cell
kv = 5	ratio of cytoplasmic to nuclear volume
x13(0) = 0.06	Initial value of NF-κB is given in cytoplasmic complex(IkBa|NFkB)

**Table:** **Chemical reactions for BDNF and TrKB signaling pathways (Module 2)**.

y(1) = BDNF + TrKB → BDNF_TrKB_clx	% Ligand Binding
y(2) = BDNF_TrKB_clx + TrKB → BDNF_TrKB2_clx	% Receptor dimerization
y(3) = BDNF_TrKB2_clx → BDNF_TrKB2*_clx	% Autophosphorylation
y(4) = BDNF_TrKB2*_clx ⇑ Int_BDNF_TrKB2*_clx	% Receptor Internalization
y(5) = Int_BDNF_TrKB2*_clx → TrKB	% Receptor Cycling
y(6) = Shc + BDNF_TrKB2*_clx → BDNF_TrKB2*_clx + Shc*	% Shc Phosphorylation
y(7) = Shc* → Shc	% Dephosphorylation Shc*
y(8) = PLC-γ + BDNF_TrKB2*_clx → BDNF_TrKB2*_clx + PLC-γ*	% PLC-γ Phosphorylation
y(9) = PLC-γ + PLC-γ_basal → PLC-γ_basal + PLC-γ*	% PLC-γ basal Phosphorylation
y(10) = PLC-γ* → PLC-γ	% Dephosphorylation of PLC-γ
y(11) = Grb2 + Shc* → Shc*-Grb2	% Grb2 binding Shc*
y(12) = Shc*-Grb2 + Gab1 → Shc*_Grb2_Gab1	% Formation of Shc*_Grb2_Gab1
y(13) = Shc*_Grb2_Gab1 + PI3K → Shc*_Grb2_Gab1_PI3K_clx	%PI3K activation by Shc_Grb2_Gab1 complex
y(14) = Ras-GTP + PI3K → Ras-GTP_PI3K	% PI3K activation by Ras-GTP
y(15) = PIP2 + Shc*_Grb2_Gab1_PI3K_clx → Shc*_Grb2_Gab1_PI3K_clx + PIP3	% Formation of PIP3
y(16) = PIP3 + PTEN → PTEN + PIP2	% Formation of PIP2
y(17) = PI3K → PI3K_basal	% Formation of basal PIP3
y(18) = PIP2 + PI3K_basal → PI3K_basal	% Basal PI3K activity
y(19) = PIP3 + PDK1 → PIP3_PDK1	% PDK1 translocation
y(20) = PIP3 + AKT → PIP3_AKT	% AKT translocation
y(21) = PIP3_AKT + PIP3_PDK1 → PIP3_PDK1 + PIP3_AKT_thr308	% Partial AKT activation (Thr308)
y(22) = PIP3_AKT_thr308 + PP2A → PP2A + PIP3_AKT	% Dephosphorylation of AKT*
y(23) = PIP3_AKT_thr308 + PIP3_PDK2 → PIP3_PDK2 + PIP3_AKT_t308_s473	%Fully AKT activation (Ser473)
y(24) = PIP3_AKT_t308_s473 + PP2A → PIP3_AKT_thr308 + PP2A	% Dephosphorylation of AKT**
y(25) = TSC1–TSC2 + PIP3_AKT_t308_s473 → PIP3_AKT_t308_s473 + TSC1–TSC2*	%Phosphorylation of TSC1,2
y(26) = TSC1–TSC2* → TSC1–TSC2	% Dephosphorylation of TSC1–TSC2*
y(27) = Rheb-GTP + TSC1–TSC2 → TSC1–TSC2 + Rheb-GDP	% Hydrolysis of Rheb-GTP by TSC1–TSC2
y(28) = Rheb-GDP → Rheb-GTP	% Conversion of Rheb-GDP to Rheb-GTP
y(29) = IP3R + IP3 → IP3RIP3	% IP3R interaction with IP3 and formation of IP3RIP3
y(30) = IP3RIP3 + IP3 → IP3R2IP3	% IP3RIP3interaction with IP3 and formation of IP3R2IP3
y(31) = IP3R2IP3 + IP3 → IP3R3IP3	%IP3R2IP3interaction with IP3 and formation of IP3R3IP3
y(32) = IP3R3IP3 + Ca2 → IP3R3IP3 + Ca(cyt)	%IP3R3IP3 interaction with Ca^2+^ and formation of IP3R3IP3 and Ca^2+^
y(33) = CaM-Ca4 + CAMKIII → CaMKIII_CaM-Ca4	% Binding of Calmodulin-Calcium to CaMKIII
y(34) = CaMKIII_CaM-Ca4 + eEF2 → eEF2thr-56 + CaMKIII_CaM-Ca4	% Phosphorylation of eEF2
y(35) = eEF2thr-56 + PP2A → eEF2 + PP2A	% Dephosphorylation of eEF2thr-56
y(36) = CaMKIII + S6K_thr-252 → CaMKIII* + S6K_thr-252	% Phosphorylation of CaMKIII
y(37) = CaMKIII + S6K_Basal → CaMKIII* + S6K_thr-252	% Basal activation of CaMKIII

**Table A3:** **Chemical species and initial concentration for Module 2**.

y1(0)	BDNF	0.1 nM
y2(0)	TrKB	0.25 μM
y3(0)	Int_BDNF_TrKB2*_clx	0.25 μM
y6(0)	Shc	0.5 μM
y8(0)	PLC-γ	0.1 μM
y9(0)	PLC-γ_basal	0.0007 μM
y11(0)	Grb2	1 μM
y12(0)	Gab1	0.7 μM
y13(0)	PI3K	0.1 μM
y15(0)	PIP2	7 μM
y16(0)	PTEN	0.27 μM
y19(0)	PDK1	1 μM
y20(0)	AKT	0.2 μM
y22(0)	PP2A	0.15 μM
y23(0)	PIP3_PDK2	0.003 μM
y25(0)	TSC1–TSC2	1 μM
y27(0)	Rheb-GTP	1 μM
y29(0)	IP3	1 μM
y33(0)	CaMKIII	0.06 μM
y34(0)	eEF2	0.5 μM
y35(0)	PP2A	0.15 μM
y36(0)	S6K	0.17 μM

**Table A4:** **The following association and dissociation rate constants for Module 2 are taken from Ref. (29–33, 37, 39–80)**.

BD = 0 (BDNF inactive)BD = 1 (BDNF active)
TR = 0 (TrkB inactive)
TR = 1 (TrkB active)
k1f = 1; k1b = 0.05
k2f = 1; k2b = 0.02
k3f = 0.02; k3b = 0
k4f = 0.01; k4b = 0
k5f = 0.001; k5b = 0.001
k6f = 0.8333; k6b = 0.3
k7f = 0.2; k7b = 0
k8f = 0.3; k8b = 0.5
k9f = 0.3; k9b = 0.5
k10f = 0.07; k10b = 0
k11f = 1; k11b = 1
k12f = 0.3; k12b = 1
k13f = 5; k13b = 0.08
k14f = 1.8; k14b = 5
k15f = 4; k15b = 4
k16f = 0.08; k16b = 5.5
k17f = 0.00068; k17b = 0.45
k18f = 4; k18b = 4
k19f = 0.7; k19b = 0.98
k20f = 45; k20b = 0.089
k21f = 0.4; k21b = 10
k22f = 4.8; k22b = 1.8
k23f = 0.8; k23b = 20
k24f = 4.8; k24b = 1.8
k25f = 10.3; k25b = 6
k26f = 0.01; k26b = 0
k27f = 0.3; k27b = 20
k28f = 0.2; k28b = 0
k29f = 100; k29b = 3
k30f = 100; k30b = 2
k31f = 100; k31b = 1
k32f = 100; k32b = 0.1
k33f = 0.99; k29b = 0.09
k34f = 2; k30b = 10
k35f = 8.8; k31b = 0.47
k36f = 1; k32b = 1
k37f = 1; k33b = 10
CaCyt = 0.1
Ca = 0.5
eCAMKIII = 0.06
S6K_thr = 0.001
S6K_Basal = 0.001

**Module 1:** **Pharmacotherapy (minocycline) activation through TNF-α in NF-κB signaling pathway**.

dx(1) = kprod-kdeg*x(1) − TN*r1*x(1)	% neutral IKK
dx(2) = TN*r1*x(1) − r3*x(2) − TN*r2*x(2)*x(8) − kdeg*x(2) − a2*x(2)*x(10) + t1*x(4) − a3*x(2)*x(13) + t2*x(5)	% free active IKK
dx(3) = r3*x(2) + TN*r2*x(2)*x(8) − kdeg*x(3)	% inactive IKK
dx(4) = a2*x(2)*x(10) − t1*x(4)	% cytoplasmic (IKK|IkBa) complex
dx(5) = a3*x(2)*x(13) − t2*x(5)	% cytoplasmic (IKK|IkBa|NFkB) complex
dx(6) = c6a*x(13) − a1*x(6)*x(10) + t2*x(5) − i1*x(6)	% Free cytoplasmic NFkB
dx(7) = i1*kv*x(6) − a1*x(11)*x(7)	% Free nuclear NFkB
dx(8) = c4*x(9) − c5*x(8)	% Cytoplasmic PTGS2 gene
dx(9) = c2 + c1*x(7) − c3*x(9)	% PTGS2 transcription
dx(10) = -a2*x(2)*y(10) − a1*x(10)*x(6) + c4a*x(12) − c5a*x(10) − i1a*x(10) + e1a*x(11)	% Free cytoplasmic IkBa
dx(11) = -a1*x(11)*x(7) + i1a*kv*x(10) − e1a*kv*x(11)	% Free nuclear IkBan
dx(12) = c2a + c1a*x(7) − c3a*x(12)	% IkB transcription
dx(13) = a1*x(10)*x(6) − c6a*x(13) − a3*x(2)*x(13) + e2a*x(14)	% Cytoplasmic(IkBa|NFkB) complex
dx(14) = a1*x(11)*x(7) − e2a*kv*x(14)	% Nuclear (IkBa|NFkB) complex
dx(15) = c2c + c1c*x(7) − c3c*x(15)	% Control gene mRNA level

**Module 2:** **We now formulate the ordinary differential equations for the above chemical reactions (Eqs 1–37)**.

1.	dy(1) = k1f* dy(1)*BD + k1f* dy(2)*BD − k1b* dy(3)
2.	dy(2) = k2f* dy(3) + k2f* dy(2)*TR − k2b* dy(3)
3.	dy(3) = k3f* dy(3) − k3b* dy(4)
4.	dy(4) = k4f* dy(4) − k4b* dy(5)
5.	dy(5) = k5f* dy(5) − k5b* dy(2)
6.	dy(6) = k6f* dy(6) + k6f* dy(4) − k6b* dy(4) + dy(7)
7.	dy(7) = k7f* dy(7)-k7b* dy(6)
8.	dy(8) = k8f* dy(8) + dy(4) − k8b* dy(4) -k8b* dy(10)
9.	dy(9) = k9f* dy(8) + k9f* dy(9)- k9b *dy(9) − k9b *dy(10)
10.	dy(10) = k10f* dy(10) − k10b* dy(8)
11.	dy(11) = k11f* dy(11) + k11f* dy(7) − k11b* dy(12)
12.	dy(12) = k12f* dy(12) − k12b* dy(13)
13.	dy(13) = k13f* dy(13) + k13f* dy(17) − k13b* dy(13)
14.	dy(14) = k14f* dy(14) + k14f* dy(17) − k14b* dy(14)
15.	dy(15) = k15f* dy(15) + k15f* dy(13) − k15b* dy(13) − k15b* dy(16)
16.	dy(16) = k16f* dy(16) + k16f* PTEN − k16b* PTEN − k16b* dy(15)
17.	dy(17) = k17f* d(17) − k17b* PI3K_basal
18.	dy(18) = k18f* dy(15) + k18f* PI3K_basal − k18b* PI3K_basal
19.	dy(19) = k19f* dy(16) + k19f* dy(19) − k19b* dy(19)
20.	dy(20) = k20f * dy(16) + k20f* dy(20) − k20b* dy(21)
21.	dy(21) = k21f* dy(20) + k21f* dy(19) − k21b* dy(19) − k21b* dy(22)
22.	dy(22) = k22f* dy(22) + k22f* PP2A − k22b* PP2A -k22b* dy(21)
23.	dy(23) = k23f* dy(22) + k23f* PIP3_PDK2 − k23b* PIP3_PDK2 − k23b* dy(24)
24.	dy(24) = k24f* dy(24) + PP2A − k24b* dy(22) − k24b* PP2A
25.	dy(25) = k25f* dy(25) + k25f * dy(24) − k25b* dy(24) − k25b* dy(26)
26.	dy(26) = k26f* dy(26) − k26b* dy(25)
27.	dy(27) = k27f* dy(27) + k27f * dy(25) − k27b* dy(25) − k27b* dy(28)
28.	dy(28) = k28f* dy(28) – k28b* dy(28)
29.	dy(29) = k29f* dy(29) + k29f*IP3 − k29b* dy(30)
30.	dy(30) = k30f* dy(30) + k30f*IP3 − k30b* dy(31)
31.	dy(31) = k31f* dy(31) + k31f*IP3 − k31b* dy(32)
32.	dy(32) = k32f* dy(32) + k32f*Ca − k32b* dy(32) − k32b*Cacyt
33.	dy(33) = k33f* dy(33) + k33f* dy(36) − k37b* dy(34)
34.	dy(34) = k34f* dy(34) + k34f* eEF2 − k34b* dy(335) − k334b* dy(34)
35.	dy(35) = k35f* dy(35) + k35f* PP2A − k35b* eEF2 − k35b* PP2A
36.	dy(36) = k36f* dy(36) + k36f* S6K_thr − k36b* dy(37) − k36b* S6K_thr
37.	dy(37) = k37f* dy(36) + k37f* S6K_Basal − k37b* dy(36) − k37b* S6K_thr
